# Ninety-six–hour starved peripheral blood mononuclear cell supernatant inhibited LA7 breast cancer stem cells induced tumor *via* reduction in angiogenesis and alternations in *Gch1* and *Spr* expressions

**DOI:** 10.3389/fimmu.2022.1025933

**Published:** 2023-02-23

**Authors:** Maryam Mehri, Reza Gheitasi, Roghayeh Pourbagher, Mohammad Ranaee, Kosar Nayeri, Seyed Mostafa Rahimi, Hamid Reza Khorasani, Hadi Hossein-Nattaj, Davood Sabour, Haleh Akhavan-Niaki, Sadegh Fattahi, Behnam Kalali, Amrollah Mostafazadeh

**Affiliations:** ^1^ Student Research Committee, Babol University of Medical Sciences, Babol, Iran; ^2^ Institute of Infectious Diseases and Infection Control, Jena University Hospital/Friedrich Schiller University, Jena, Germany; ^3^ Cellular and Molecular Biology Research Center, Health Research Institute, Babol University of Medical Sciences, Babol, Iran; ^4^ Department of Pathology, School of Medicine, Health Research Institute, Babol University of Medical Sciences, Babol, Iran; ^5^ Department of Cancer Medicine, Cell Science Research Center, Royan Institute for Stem Cell Biology and Technology, Academic Center for Education, Culture and Research (ACECR), Babol, Iran; ^6^ Department of Stem Cells and Developmental Biology, Cell Science Research Center, Royan Institute for Stem Cell Biology and Technology, Academic Center for Education, Culture and Research (ACECR), Tehran, Iran; ^7^ Immunology Department, Mazandaran University of Medical Sciences, Sari, Iran; ^8^ Department of Genetics, Faculty of Medicine, Babol University of Medical Sciences, Babol, Iran; ^9^ North Research Center, Pasteur Institute of Iran, Amol, Iran; ^10^ Department of Medicine II, Klinikum Grosshadern, Ludwig Maximilian University (LMU) University, Munich, Germany

**Keywords:** breast cancer stem cells, LA7, adaptive immunity, serum starvation, nano-LC-ESI-MS/MS, cancer development, immunohistochemistry

## Abstract

**Introduction:**

The microenvironment of solid tumors such as breast cancer is heterogeneous and complex, containing different types of cell, namely, cancer stem cells and immune cells. We previously reported the immunoregulatory behavior of the human immune cell in a solid tumor microenvironment-like culture under serum starvation stress for 96 h. Here, we examined the effect of this culture-derived solution on breast cancer development in rats.

**Method:**

Ninety-six–hour starved PBMCs supernatant (96 h-SPS) was collected after culturing human PBMCs for 96 h under serum starvation condition. Breast cancer stem cells, LA7 cell line, was used for *in vitro* study by analyzing gene expression status and performing cytotoxicity, proliferation, scratch wound healing assays, followed by *in vivo* tumor induction in three groups of mature female Sprague Dawley rats. Animals were treated with 96 h-SPS or RPMI and normal saline as control, *n* = 6 for each group. After biochemical analysis of iron, lactate, and pH levels in the dissected tumors, Ki67 antigen expression, angiogenesis, and necrosis evaluation were carried out. Metabolic-related gene expression was assessed using RT-qPCR. Moreover, 96 h-SPS composition was discovered by Nano-LC-ESI-MS/MS.

**Results:**

96 h-SPS solution reduced the LA7 cell viability, proliferation, and migration and *Gch1* and *Spr* genes expression *in vitro* (*p*< 0.05), whereas stemness gene *Oct4* was upregulated (*p*< 0.01). The intracellular lactate was significantly decreased in the 96 h-SPS treated group (*p =* 0.007). In this group, *Gch1* and *Spr* were significantly downregulated (*p*< 0.05), whereas the *Sox2* and *Oct4* expression was not changed significantly. The number of vessels and mitosis (Ki67^+^ cells) in the 96 h-SPS–treated group was significantly reduced (*p* = 0.024). The increased rate of necrosis in this group was statistically significant (*p* = 0.04). Last, proteomics analysis revealed candidate effectors’ components of 96 h-SPS solution.

**Conclusion:**

96 h-SPS solution may help to prevent cancer stem cell mediated tumor development. This phenomenon could be mediated through direct cytotoxic effects, inhibition of cell proliferation and migration in association with reduction in *Gch1* and *Spr* genes expression, angiogenesis and mitosis rate, and necrosis augmentation. The preliminary data obtained from the present study need to be investigated on a larger scale and can be used as a pilot for further studies on the biology of cancer development.

## 1 Introduction

The world’s leading cause of cancer death in women is breast cancer (BC) (1). The prevalence of BC is increased to 6% in women over the age of 70 years (2). Recent studies indicate cancer stem cells (CSCs) may cause most cancers (3). CSC hypothesis relies on two bases: (a) dysregulation of the signaling pathways leads to the development of cancers in the tissue-specific stem or progenitor cells or (b) epithelial cells may undergo reprogramming to form CSCs (4). As a result, tumors contain a cellular component that preserves the fundamental characteristics of stem cells, such as the capacity for self-renewal, treatment resistance, and tumor recurrence, which all promote tumorigenesis and cell differentiation (5). The CSC theory has important implications to understand the molecular basis of complexity and the early diagnosis, prevention, and treatment of BC (6). Effective targeting of CSCs may serve as a preventive strategy for cancer development.

In humans, most cancers consist of carcinomas (7), which can be the outcome of the interaction between cancer cells, stromal cells, and immune cells (8). The niche of solid tumors is a heterogeneous environment consisting of an extracellular matrix, resident fibroblasts, endothelial cells, and immune cells may also be involved in cancer progression (9). In the niche of the tumor, contributing cells are exposed to metabolic stress; however, immune cells must keep their protective functions in such conditions by synthesizing different types of proteins, that is, cytokines, chemokines, and other effector molecules essential to perform their defensive activities (10). To date, the effect of nutrient starvation on immune cell metabolism is not known. Nevertheless, immune cells in the tumor microenvironment can play inhibitory and/or stimulatory role in tumor development (11) type 2 macrophages (M2), type 2 neutrophils (N2), and regulatory T cells (Treg) are known as suppressor cells in the tumor microenvironment and modulate the immune system, whereas cytotoxic T cells (CTL), Natural Killer (NK) cells, and T helper1 (Th1) cells are recognized as anti-tumor cells (12, 13). Similar to other cells, CSCs also rely on metabolism for survival and growth (14). In recent years, it has come to light that Sepiapetrin reductase (SPR) and GTP cyclohydrolase I (GCH1) play a crucial role in the metabolic processes of cancer cells (15). Although the contribution of these two enzymes to generate reactive oxygen species (ROS) is well described, GCH1 is a rate-limiting enzyme in the metabolic pathway that generates tetrahydrobiopterin (BH4) (16). BH4 is a critical cofactor for nitric oxide synthase (NOS) (17). Reduced GCH1 activity can result in tumor vascularization and lowering of NOS, which can produce superoxide (18). The expression of the GCH1 gene in cancer is linked to poor prognosis and increased cancer grade (19).

Likewise, genes such as *Octamer-binding transcription factor 4 (Oct4) and SYR-box transcription factor 2 (Sox2)* are linked to stemness (20). OCT4 and SOX2 transcription factors are necessary for the generation of induced pluripotent stem cells (iPSCs) and the maintenance of embryonic stem cells (ESCs) (21). Depending on the arrangement of their respective DNA-binding regions, OCT4 and SOX2 interact and bind to DNA in a variety of configurations (22). It is renowned that they play a crucial part in the process of preserving the pluripotency characteristics of CSCs and contribute to the development, growth, and metastasis of tumors (23).

Studying the behavior of the immune cells in a special condition in microenvironments of solid tumors such as culture under nutrient starvation stress could improve tumor biology understanding (24–26). Previously, we showed that human peripheral blood mononuclear cells (PBMCs), which were cultured under serum starvation condition for 96h, downregulated the expression of an important immunoregulatory molecule, that is, HLA on their surface while more than 70% of the cells were alive in annexin V/propidium iodide apoptosis assay (27). In another study with human PBMCs, we examined the effects of such metabolic stress at different time points, on transforming growth factor -β expression, CD4^+^ CD25^+^ CD127^-^ FOXP3^+^ T-regulatory cell frequency, and expression of several type of microRNAs involved in immunity and immunometabolism. We observed that the maximum levels of alteration, almost in all studied variables, occurred in 96h starved cells group (28). The mass spectrometry analysis of the cell culture supernatant of 96 h-SPS group revealed a proteome with more than 200 proteins with different biological functions including cell metabolism and cell death regulation (published patent under international application no. PCT/EP2020/087239). In the present study, we aimed to evaluate the *in vitro* and *in vivo* impacts of 96 h-SPS on biological features of LA7 cell, a rat mammary gland cancer stem cell line and LA7-induced solid tumor development as well as to understand the mechanisms underlying these effects. Here, we got advantage of the immune cells secreted compounds under serum starvation cultured *in vitro* to examine the application of these compounds in cancer immunotherapy through targeting of CSCs.

## 2 Material and methods

### 2.1 Preparation of 96 h-SPS solution

Blood samples of seven healthy volunteer donors (six women and one man, age ± SD: 26 years ± 1.7) were collected in heparinized sterile tubes (Golden Vac, Turkey). Ficoll-Plaque (Capricorn Scientific, Meuspath, Rheinland-Pfalz, Germany) gradient centrifugation was used for PBMCs isolation by centrifugation at 450*g* for 30 min. The PBMCs layer was washed twice using phosphate-buffered saline (PBS) by centrifugation at 350*g* for 5 min. Then, 7 × 10^6^ PBMCs were cultured in a 75 cm^2^ cell culture flask containing 12 ml of starvation media (glucose-containing serum-free Roswell Park Memorial Institute 1640 medium (RPMI 1640), (Biowest, France) 100 U/ml penicillin, and 100 µg/ml streptomycin (Sigma, Germany) for 96h at 37°C with 5% CO2 saturation. Then, the cells were collected and centrifuged at 350*g* for 5 min and cell culture supernatant (96 h-SPS) was removed and stored at −20°C for further use.

### 2.2 Cytotoxicity (MTT assay (3-(4, 5- dimethylthiazolyl-2)-2,5 diphenyltetrazolium bromide)), migration (scratch test), and proliferation (CFSE-staining) assays

#### 2.2.1 MTT assay

The MTT assay was performed based on the method that has been already published (29). Briefly, 1.2 × 10^4^ LA7 cells were cultured in a 96-well culture plate as five repeats in Dulbecco’s Modified Eagle Medium (DMEM) (Gibco), supplemented with 10% fetal bovine serum (FBS, Sigma, Las Vegas (LA), USA) and antibiotics (penicillin/streptomycin) 1% at 37°C with 5% CO2. After 24h incubation, cell confluency reached 70% and cells were washed twice with PBS and then 96 h-SPS and control solutions were added to indicated wells. RPMI and RPMI + FBS 10% (complete medium) were used as vehicle- and cell growth–positive control, respectively. The cell incubation was continued further for 24h and, after then, the wells were washed twice with PBS and the cells were incubated with 50 μl of 5 mg/ml MTT (3-(4, 5- dimethylthiazolyl-2)-2,5-diphenyltetrazolium bromide) (Life biolab, Germany) solution in PBS for 4h at 37°C. The supernatants were then removed and replaced by 150 μl of dimethyl sulfoxide (DMSO) and pipetting was performed thoroughly to dissolve formazan crystals. Then, the optical density (OD) was determined at 570 nm. The percentage of viability was calculated by the following formula: Viability (%) = (OD test)/(OD control) × 100.

Before adding the MTT solution to the wells, the cells were evaluated morphologically under inverted microscope (Olympus, Japan) and microphotographs were captured by camera (Olympus, Japan) equipped with GP25 software.

#### 2.2.2 Scratch test

The scratch test was carried out by a method which we have previously reported (29). To this end, 1 × 10^5^ LA7 cells were cultured as triplicate in a 24-well cell culture plate under standard conditions for animal cell culture, as described above, until reaching 100% confluency. Then the cell’s monolayer was vertically scratched using a 100-µl micropipette tip and washed twice with PBS. After then, 96 h-SPS and control solutions were added and incubation was continued for 48h. RPMI and RPMI + FBS 10% were considered as vehicle- and cell migration positive control, respectively. The required microphotographs were captured at 0h and after 24h and 48h using an inverted microscope (Olympus, Japan) equipped with a camera coupled to GP25 software. Finally, the empty area in each well was analyzed with image J software (NIH, Rockville Pike, Bethesda, USA).

#### 2.2.3 CFSE staining

The proliferation rate of cells was assessed using 5-(and 6)-carboxyfluorescein diacetate succinimidyl ester (CFSE), (eBioscience, USA). Briefly, cells were washed twice with PBS. Then 5 × 10^6^ cells were suspended in 1 ml of PBS. 5 μM CFSE was dispensed into cell suspension, mixed, and incubated for 10 min at room temperature in the dark. CFSE staining was stopped by adding cold complete medium and incubating on ice for 5 min. Cells were washed three times with complete medium. 6 × 10^4^ CFSE stained cells were fixed in formalin 1% + FBS 10% and kept at 4°C until use. These cells were used as stained cell control for day 0 in flow cytometry analysis.

Then, the rest of CFSE stained cells were cultured in complete medium in a 24-well cell culture plate as 2 × 10^4^ cells/well. The same number of non-labeled cells was seeded as unstained control cells. After 24h, wells were washed twice with PBS and then incubated with 96 h-SPS as well as above mentioned control media for 24h and 48h. The 24h incubated cells were detached by trypsin 0.05% + ethylene diamine tetra acetic acid (Gibco), centrifuged at 450*g* for 5 min and the cell pellet of each group was fixed with the same volume of formalin 1% + FBS 10% and kept at 4°C until flow cytometry analysis. After harvesting the 48h incubated cells and performing formalin fixation, all collected stained cells (day 0, 24h, and 48h) and unstained control cells were analyzed simultaneously by flow cytometry (Partech, Germany). Almost 1 × 10^4^ events were analyzed by flow cytometry instrument except for the cells which were incubated with 96 h-SPS solution for 48h, because of low number of remained treated cells.

Before cells were harvested for flow cytometry analysis, the cells were evaluated morphologically in different groups under inverted light microscope (Olympus, Japan) and microphotographs were prepared by camera (Olympus, Japan) equipped with GP25 software.

### 2.3 Tumor induction and treatment with 96 h-SPS

#### 2.3.1 Induction

Tumor induction was performed as previously described (30). To this end, CSCs (LA7) were cultured under standard animal cell culture conditions to reach a confluency of 70–80%. Then, using viable cells, tumor induction was performed in three groups (96 h-SPS, RPMI, and normal saline) of six mature female Sprague Dawley rats. LA7 cells (6 x 10^6^/cells) in normal saline were injected into the rat breast fat pad subcutaneously in all groups.

#### 2.3.2 Treatment

96 h-SPS was administered to the first group, RPMI to the second, and normal saline to the third group. Treatment solutions in each group were injected as 300 μl daily in three areas around the tumor until the 12^th^ day. The animals’ weight was recorded almost daily, and tumor length and width were measured with a caliper when they were well palpable. Tumor volume was calculated with the following formula:


$$volume=\frac{width*(length^{2})}{2}$$

Tumor lesions were removed on the 13^th^ day and pathological and biochemical examinations were performed; also, the expression of targeted genes was assessed by polymerase chain reaction (PCR) as described below.

### 2.4 Identification of proteins

Nanoscale liquid chromatography coupled to tandem mass spectrometry (Nano-LC-ESI-MS/MS, EASY-nLC1000 – LTQ Orbitrap XL, Thermo Scientific) was used to evaluate 96 h-SPS. The procedure for sample preparation was as follows:

#### 2.4.1 Lyophilization

One ml of each sample (approximately 20 μg protein) was transferred into 2-ml Protein LoBind^®^ Tubes (Eppendorf, Germany) with pierced lids and frozen for 66h at – 80°C. Subsequently, the frozen samples were vacuum dried in a lyophiliser overnight. The dried samples were each resolved in 50-μl buffer (8 M urea/0.4 M NH4HCO3, pH 8.5).

#### 2.4.2 Reduction and alkylation

Both samples were reduced with 5 μl of 45 mM dithiothreitol for 30 min at 55°C and alkylated with 5 μl of 100 mM iodoacetamide for 15 min at room temperature in the dark. Samples were prevented from over-alkylation by adding another 5 μl of 45 mM dithiothreitol.

#### 2.4.3 Enzymatic digestion

The sample solution was diluted to 2 M urea/0.1 M NH4HCO3 with HPLC grade water (VWR). Digestion with mass-spectrometry grade trypsin (Serva, porcine) was performed at 37°C overnight. Enzymatic digestion was performed based on the valid version of the SOPH-VED-001 “EnzymatischeSpaltung.” The sample was acidified to 1% TFA, and 10 μl of the digestion product was used for the Nano-LCESIMS/MS analysis.

#### 2.4.4 Nano-LC-ESI-MS/MS

HPLC separation was done using an EASY-nLC1000 (Thermo Scientific, USA) system with the following columns and chromatographic settings: The peptides were applied to a C18 column (Acclaim^®^ PepMap 100 pre-column, C18, 3 μm, 2 cm x 75 μm Nanoviper, Thermo Scientific, USA) and subsequently separated using an analytical column (EASY-Spray column, 50 cm x 75 μm ID, PepMap C18 2 μm particles, 100 Å pore size, Thermo Scientific, USA) by applying a linear gradient (A: 0.1% formic acid in water, B: 0.1% formic acid in 100% ACN) at a flow rate of 280 nl/min. The gradient used was 1–25% B in 120 min, 25-50% B in 10 min, 84% B10 min. Mass-spectrometric analysis was done on an LTQ Orbitrap XL mass spectrometer (Thermo Scientific, USA), which was coupled online to the HPLC system. The mass spectrometer was operated in the so-called “data-dependent” mode where after each global scan the five most intense peptide signals are chosen automatically for MS/MS analysis. Nano-LC HPLC separation and online-coupled ESI-MS/MS measurement were performed according to the valid versions of the SOPs:P-EXT-011 “Auftrennung und Analyse von Peptidmischungenmit der nanoflow-HPLCAnlageEksigentNanoLC-Ultra ‘P-EXT-012’ MS/MS Analyse von Peptidenmit dem Massenspektrometer Orbitrap XL.”

### 2.5 Biochemical analysis

#### 2.5.1 Lactate and pH measurement

The tumor lactate level was determined using a fluorometric lactate enzymatic test kit (biorexfars, Iran) after the tumors were homogenized in 500 ml of distilled water using a LABTron homogenizer (LABTron, Korea). The actual pH of each sample was then tested using a pH meter (Mettler Toledo, Switzerland) and the following formula, and the findings based on the weight of the tumor were acquired.


$$\left[H^{+}\right]\;=10^{-PH}/weight\;=X\;-\;Log\;X\;=\;pH$$

#### 2.5.2 Iron measurement

Iron was measured by an atomic absorption spectrometer (GBC932 AA, Australia), according to manufacturer instructions. Briefly, 10 mg of tissue was placed in a clean glass container and was dried at 10°C overnight. Next, dried tissue weight was measured with an accurate scale, and the weighed dry tissue was placed in a tall glass tube containing 400 μl of concentrated H_2_O_2_ and 200 μl of nitric acid (digest solution, Merck, Germany). Then, it was placed in a pan at 95°C for 25 min to obtain a uniform solution, and the volume of solution inside the tube was topped up to 5 ml with distilled water. The digestion solution was used as a blank. In the last step, using the formula below, the amount of raw solution of the device and the amount of iron was calculated.


X = C × 5.56 × m

### 2.6 Real-time quantitative polymerase chain reaction

As previously described by Fattahi et al. (31), the primers of the four genes and universal probe were designed using the AlleleID 6.0 (**Supplementary Material Table S1**). Total RNA was extracted using standard conditions according to the kit instructions from LA7 cultured cells or tumor samples. For *in vitro* study, 1 × 10^5^ LA7 cells were cultured in DMEM (Gibco) + FBS 10% (Biosera), antibiotics (penicillin/streptomycin) 1% at 37°C with 5% CO_2_, in three independent 25 cm^2^ cell culture flasks (SPL, Korea) for each group; [complete medium (RPMI + FBS 10%), RPMI, and 96 h-SPS]. After 24h, the flasks were washed twice with PBS, and then DMEM + FBS 10% was replaced by RPMI + FBS 10%, RPMI alone, and 96 h-SPS solution, respectively, in indicated groups. Then, the cell’s total RNA was extracted by an RNA extraction kit (Parstous, Iran). The quantity and quality of extracted RNA were checked using a Nanodrop spectrophotometer (Thermo Scientific NanoDrop 2000/2000c, USA) and agarose gel electrophoresis. Then, the expression levels of abovementioned genes (*Sox2*, *Oct4*, *Gch1*, and *Spr*) were analyzed. The glyceraldehyde 3-phosphate dehydrogenase *(Gapdh)* gene was considered as housekeeping. Briefly, cDNA was synthesized using mRNA-specific primer and RevertAid M-MLV Reverse Transcriptase (Yektatajhiz, Iran) (31). The mRNA levels of transcripts were performed with a universal Taqman probe using ABI 7300 real-time PCR system (Step one plus, Applied Biosystem, USA).

### 2.7 Hematoxilline-Eosin and immunohistochemistry staining

All tissue samples were embedded in paraffin after being fixed in formalin 10%. The paraffin blocks were sectioned at 5-μm thickness, and the tissues were stained with hematoxilline (H) & eosin (E) or with monoclonal antibody specific to Ki67 antigen (Dako, Denmark, Clone MIB-1), according to the manufacturer’s instructions (Novocastra, UK). Giemsa staining was used as a standard method to identify mast cells in tissue sections. The presence of necrosis and the number of mast cells and inflammatory cells were determined. Mitotic cells and blood vessels were counted in 10 and 3 high-power fields (HPF), respectively, by an expert pathologist using a light microscope (Olympus BX41, Japan). The data were presented as absence or presence of necrosis and for other indicated data as mean ± standard deviation (SD).

### 2.8 Statistical analysis

To examine normality and multiple comparisons, Kolmogorov Smirnov and one-way ANOVA with Tukey test were used, respectively. To evaluate correlation between variables, Pearson correlation coefficient was determined. Data were analyzed by SPSS software version 23, and graphs were prepared on GraphPad prism V.6.0.

The data are presented as mean ± standard error mean (SEM) or standard deviation (SD). *p*-value less than 0.05 was considered as a significant level statistically.

## 3 Result

### 3.1 *In vitro* inhibitory effect of 96 h-SPS on LA7 cells viability, migration, proliferation, and metabolism

To evaluate the effect of 96 h-SPS solution on LA7 CSC mainstay biological features, first, we determined the cell viability, cell migration, and proliferation status, and investigated the expression of *Gch1*, *Spr*, *Sox2*, and *Oct4* genes, which are involved in cell metabolism and cell stemness using an *in vitro* cell culture system.

#### 3.1.1 96 h-SPS cytotoxicity effect on LA7

**Figure 1:**
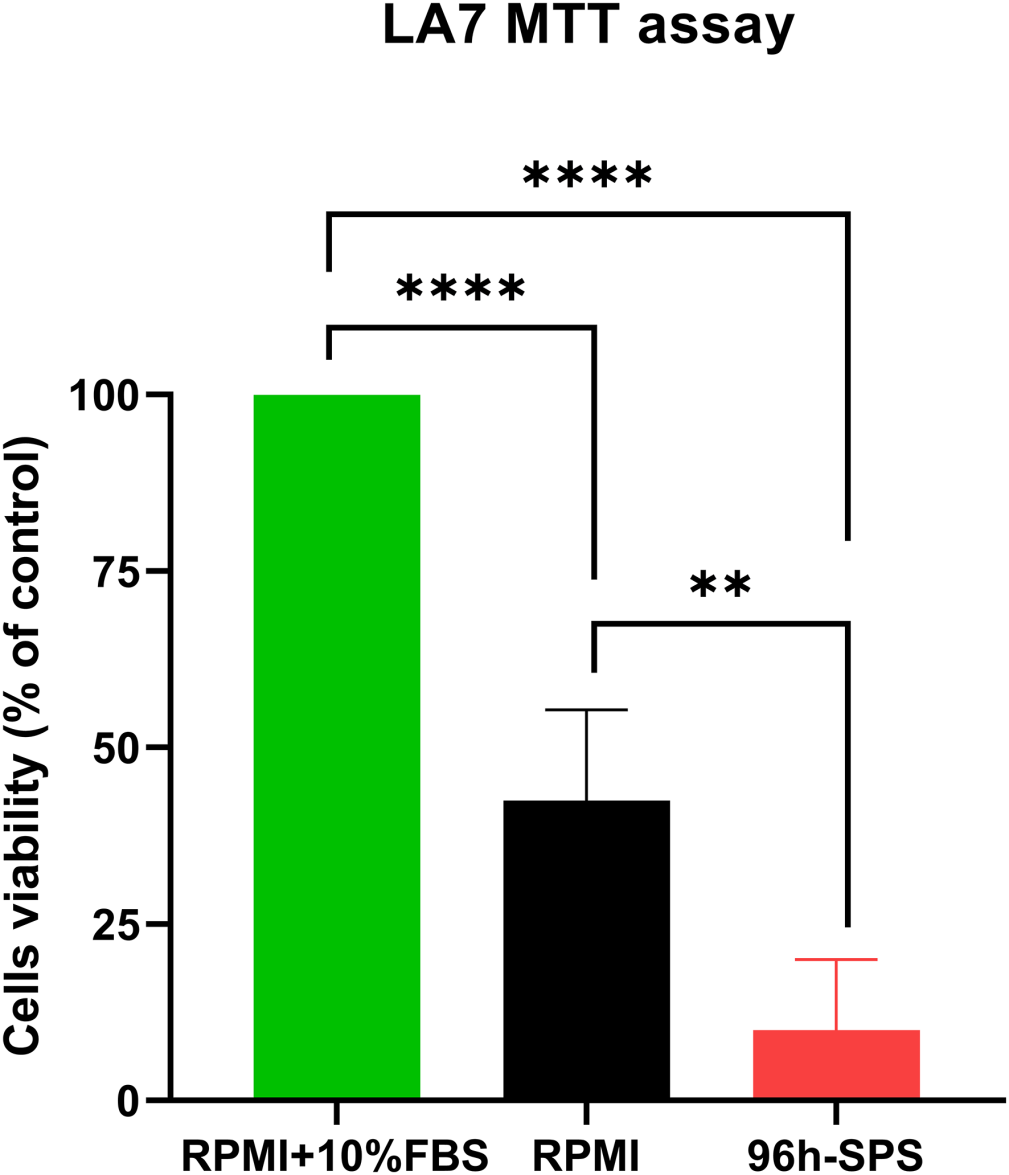
MTT assay showing the cytotoxicity of different treatments on the LA7 cell line. The 96 h-SPS treated cells showed the lowest level of viability compared with the RPMI group, *p<* 0.01, and positive control (RPMI + 10% FBS), *p<* 0.0001. Data expressed as mean ± standard error mean (SEM), bars indicate the SEM. In each panel. **p < 0.01, ****p < 0.0001.[1].

The prevalence of dying cells was higher in 96 h-SPS solution treated LA7 cells population after 24h incubation in comparison with complete medium control group (**Supplementary Material Figure S1C**). The swelling cells with round nucleus were considered as dying cells, whereas the shiny cells with spindle or polygonal morphology with non-obvious nucleus appeared as live cells. In the RPMI group, the prevalence of the dying cells was higher than that of the cells cultured in complete medium for 24h, albeit with less number of dying cells compared with the test group (**Supplementary Material Figure S1B**). This morphological finding was quantitatively confirmed by an MTT assay. The 96 h-SPS solution significantly reduced the cell’s viability (%) in comparison with the complete medium group (*p =* 0.0001) or RPMI group (*p =* 0.004) (**Figure 1**). The morphological changes observed in LA7 cells under treatment with 96 h-SPS can be explained by the MTT assay findings that indicate toxicity of 96 h-SPS solution for LA7 CSCs.

#### 3.1.2 The effect of 96 h-SPS on LA7 cells migration

**Figure 2:**
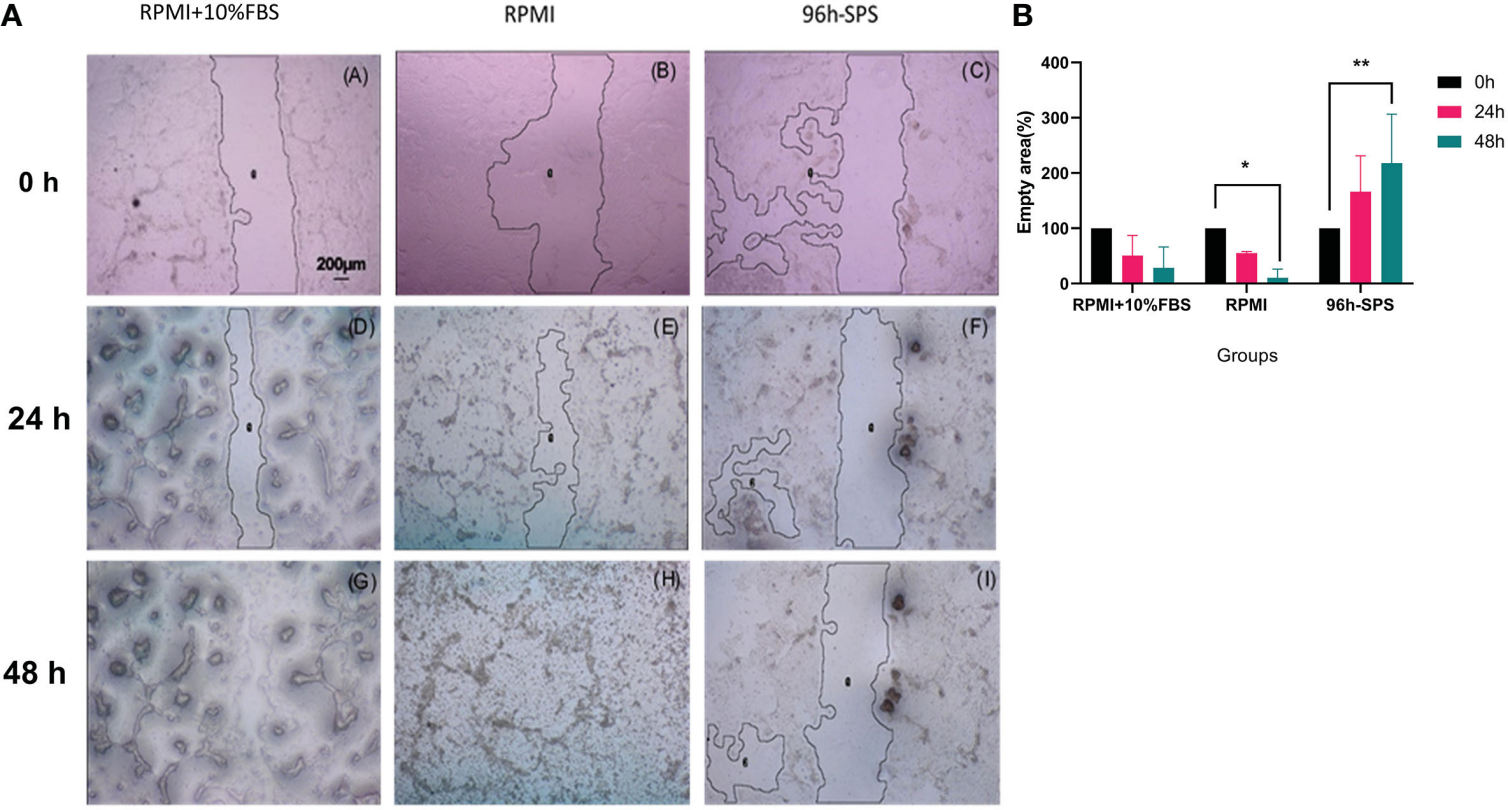
**(a)** LA7 scratch test photomicrographs. Cells culture plate scratched by 100-μl micropipette tip and exposed to the three indicated solutions for 24h and 48h. These data showed that the indicated 96 h-SPS group were able to inhibit the LA7 cells migration **(C, F, I)**, whereas the empty area was almost not observed in complete medium **(A, D, G)**, and RPMI group **(B, E, H)** after 48h. Magnification: 40×. By using image J software (NIH, USA), the relative empty area was calculated. **(b)** Quantification of the indicated empty area of LA7 cell migration under the influence of complete medium, RPMI, and 96 h-SPS group after 0h, 24h, and 48h cell incubation. The 96 h-SPS–treated LA7 cells showed a significant reduction in cell migration in comparison with RPMI. The empty area after 48h of cell incubation was significantly increased in this group compared with the initial empty area at 0h (*p* = 0.006), whereas this area was significantly decreased in RPMI group (*p* = 0.036). Data expressed as mean ± SD (*n* = 5). Bars indicate the SD. In each panel, *p< 0.05, **p< 0.01.

To evaluate the effects of complete medium, RPMI, and 96 h-SPS on migration of LA7 cells, a scratch test was carried out. According to our observation, 96 h-SPS solution was able to inhibit the cells migration during 24h and 48h incubation with LA7 cells (**Figures 2[a]-C, F, I**). However, in the complete medium and RPMI group, the empty area was almost filled after 48h (**Figures 2[a]-B, G, H**).

The data generated through scratch test empty area analysis by image J software confirmed these morphological findings. The 96 h-SPS solution treated cells exhibited a significant reduction in cell migration in comparison with RPMI alone. Empty area (%) after 48h of cell incubation was significantly increased in this group when compared with its empty area (%) at 0h (*p =* 0.006), whereas this value was significantly decreased in RPMI group (*p =* 0.036), (**Figure 2b**). Moreover, the inhibitory effects of 96 h-SPS on LA7 cells migration can be shown mathematically by calculating the Pearson coefficient of correlation between duration of cells incubation and empty area (%) in scratch assay.

Pearson correlation value (*r*) in 96 h-SPS group was + 0.99, *p<* 0.001 (**Supplementary Material Figure S2A**). In contrast, in the RPMI group, there was an utmost inverse correlation between time and empty area, *r* = -1, *p*< 0.0001 (**Supplementary Material, Figure S2B**). Similar to the RPMI group, this correlation was strongly negative in the complete medium group *r* = −0.97, *p =* 0.01 (**Supplementary Material Figure S2C**).

#### 3.1.3 The effect of 96 h-SPS on LA7 cells proliferation

CFSE staining provided more evidence for anti-cancer effects of 96 h-SPS. As it can be seen in **Supplementary Material Figure S3A**, after 24h of cell incubation, this solution postponed the cell proliferation in comparison with complete medium or RPMI alone. After 48h, the post-centrifugation cells pellet sharply decreased in 96 h-SPS treated group (data not shown). This reduction in cell number exhibited itself as a curve with the smallest area in flow cytometry plot, which was drawn by cell number against the intensity of fluorescent light emitted by CFSE stained cells (**Supplementary Material Figure S3B**). In all groups, we initially seeded the same number of stained cells and all steps of cell culture and centrifugation conditions or flow cytometry analysis were identically performed. The morphological findings confirmed these flow cytometry findings (**Supplementary Material Figures S4A–F**). These findings also provided more support for the data originated from migration analysis by scratch test.

#### 3.1.4 The effect of 96 h-SPS on LA7 cells metabolism and stemness

**Figure 3:**
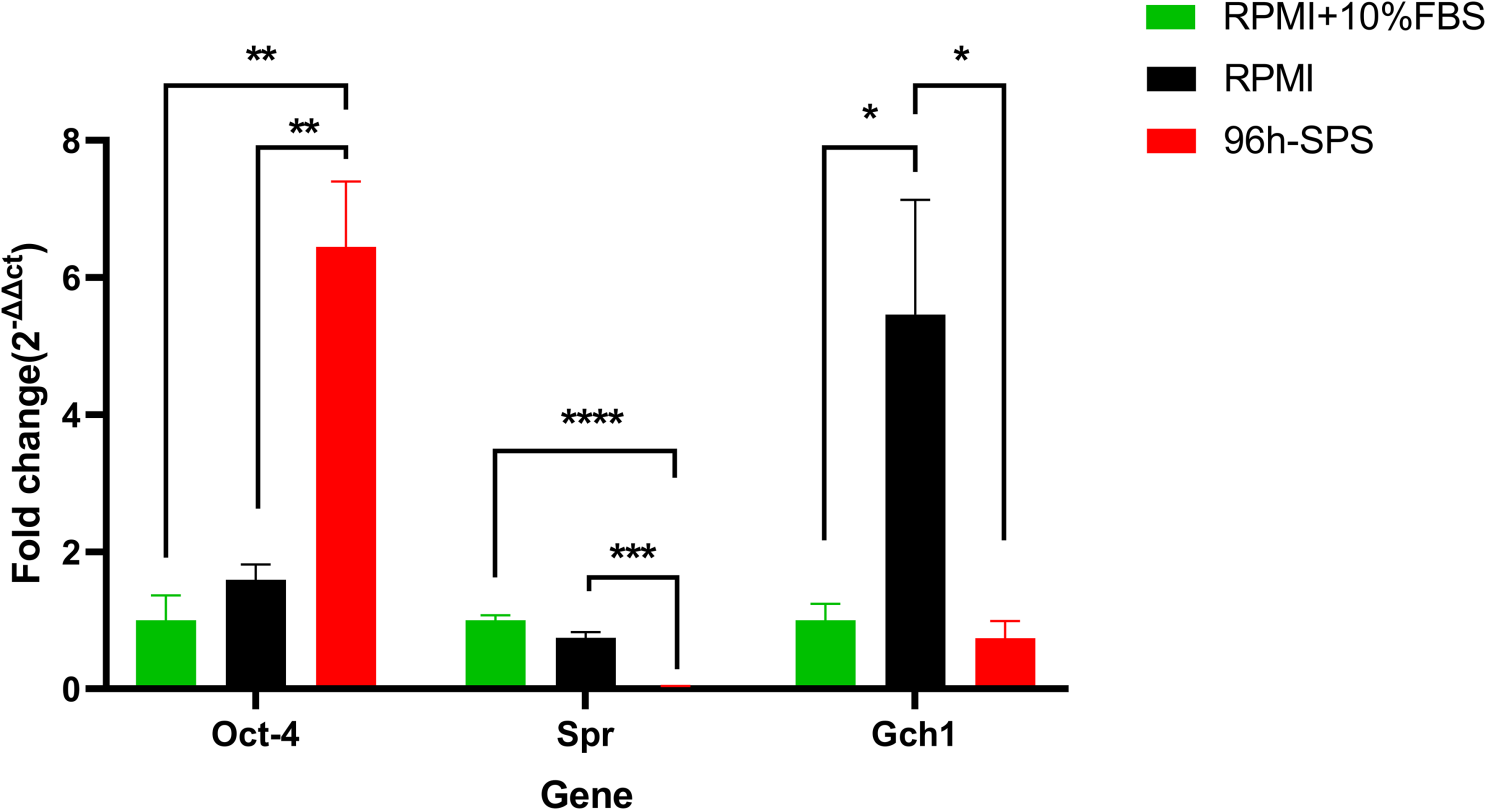
Gene expression of *Spr*, *Gch1*, and *Oct*4 in LA7 cells exposed to complete medium (RPMI + 10% FBS), RPMI, and 96 h-SPS *in vitro*. *Gapdh* was considered as an internal control. In comparison with two other solutions, 96 h-SPS–treated cells upregulated the Oct-4 gene expression, *p<* 0.01. However, this solution was significantly able to downregulate the *Spr* gene expression when compared with complete medium, *p<* 0.0001, and RPMI groups, *p<* 0.001. Data expressed as mean ± SD, bars indicate the SD. In each panel. *p < 0.05, **p < 0.01, ***p < 0.001, and ****p < 0.0001.

*Spr* and *Gch1* have some pivotal roles in the metabolic processes of cancer cells (32, 33); thus, we investigated these genes expression in the present study. *Spr* m-RNA level was significantly reduced in 96 h-SPS solution treated cells compared with the cells cultured in complete medium for 24h (*p<* 0.0001) or in RPMI alone (*p =* 0.0006) (**Figure 3**). In contrast, RPMI alone was able to increase *Gch1* expression *when* compared with the complete medium, whereas the 96 h-SPS solution prevented this alteration in *Gch1* gene expression (**Figure 3**).

To investigate the effects of 96 h-SPS solution on stemness potential of LA7 CSCs, we determined the expression of *Oct4* and *Sox2* in these cells after 24h incubation with this solution and control solutions. Surprisingly, in comparison with RPMI and complete medium, 96 h-SPS solution enhanced *Oct4* gene expression significantly *p<* 0.01 (**Figure 3**).

Because, in our real-time PCR analysis, the *Sox2* gene was not expressed at detectable level in any of complete medium or RPMI-treated flasks, we were not able to compare three indicated groups statistically in context of this gene expression. However, cross threshold (ct)values for 96 h-SPS–treated cells, cultured in three independent 25 cm^2^ flasks were detected as follows: (35.65), (38.52, undetected), and (40.65, undetected). In this PCR analysis, two PCR tubes for each flask and three independent flasks for each indicated group were analyzed. The *Gapdh* gene expression was almost at the same levels in different groups. The ct-value for this gene in complete medium group was obtained as (20.99, 20.84), (21.32, 20.97), and (21.93,21.7).This value in RPMI group was (21.35, 21.64), (23.05, 22.97), and (20.84,20.92) and in 96 h-SPS treated group was (20.41, 20.41), (20.83, 20.81), and (21.52,21.24). Thus, it could be concluded from these data that 96 h- SPS could weakly enhance *Sox2* gene expression.

### 3.2 *In vivo* inhibitory effect of 96 h-SPS on LA7 cancer stem cells induced tumor in rat

To evaluate the effect of 96 h-SPS solution on LA7 CSCs induced tumor, we determined main biological features such as the tumor growth, cytotoxicity, biochemical and pathological changes, and investigated the expression of metabolic and stemness related genes using an *in vivo* tumor study system. Moreover, we identified the protein composition of 96 h-SPS solution.

#### 3.2.1 Tumor size measurement

**Figure 4:**
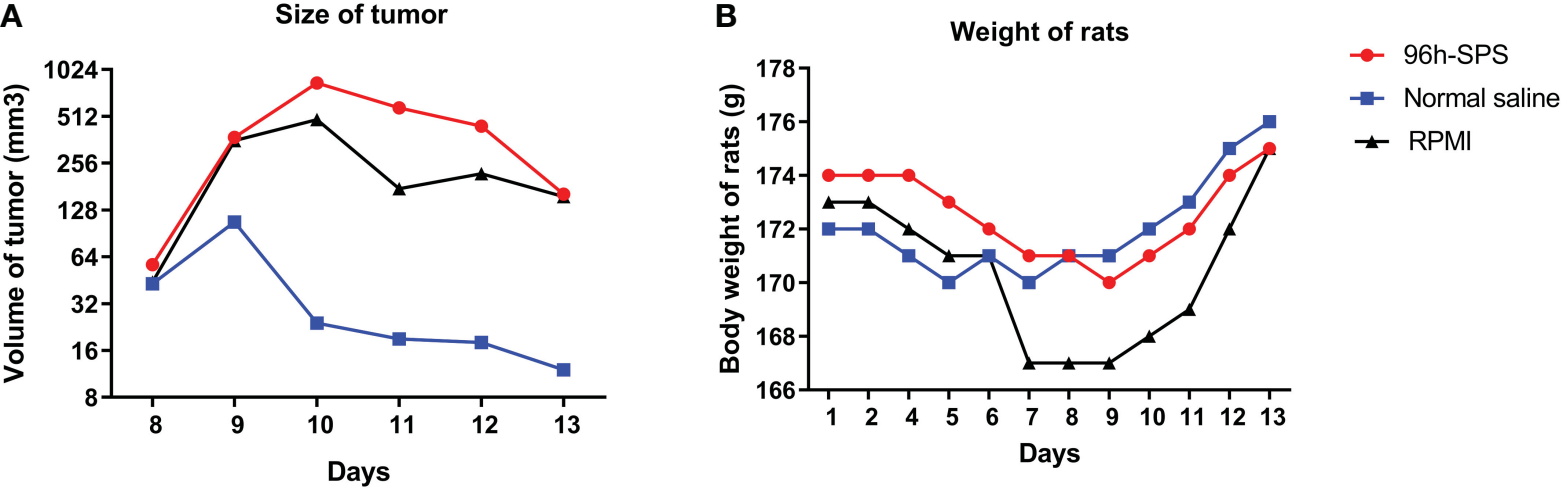
Tumor sizes and rats total body weights. **(A)** The size of tumors at different time points and **(B)** rat’s total body weight in all groups.

**Figure 5:**
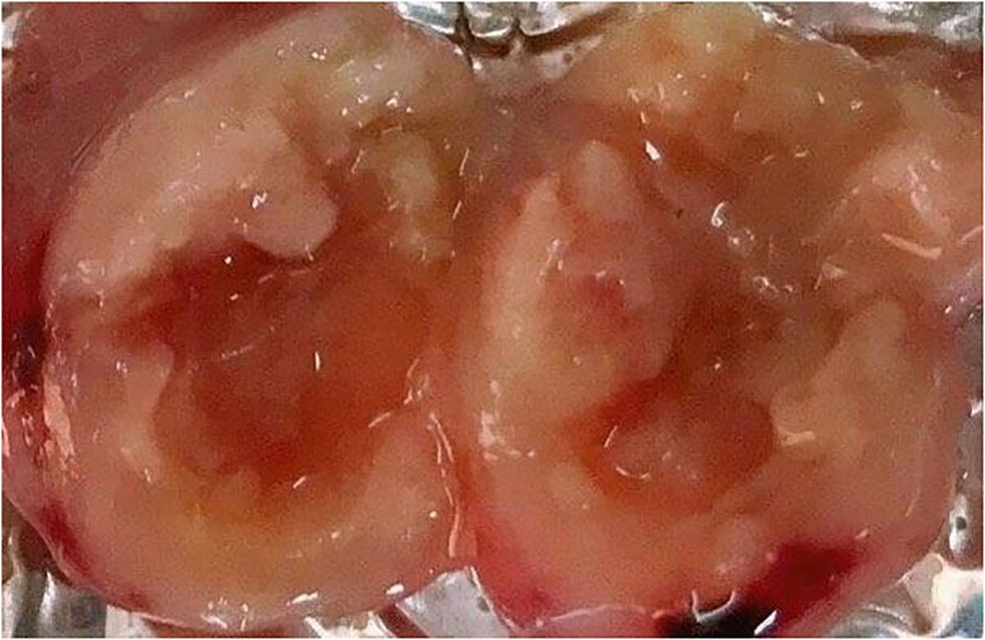
The tumor’s inner part removed from animal treated with 96 h-SPS solution.

To obtain an overall view about the effects of 96 h-SPS solution on LA7-induced tumor growth, the size and rigidity of the tumors were evaluated and animals’ weight was recorded during this study. No tumor was developed in one of the 96 h-SPS–treated rats. The size of other developed tumors treated with 96 h-SPS solution was compared with normal saline treated animals 8–13 days after LA7 cells were injected orthotopically (**Figure 4A**, *n* = 6). Surprisingly, tumor sizes were larger in the 96 h-SPS–treated group compared with normal saline-treated control (*p =* 0.027). However, contrary to this discrepancy, 96 h-SPS–treated tumors appeared as a very soft tissue with large cavities in the central part, which were filled with creamy fluids (**Figure 5**). Because different types of cell culture medium can have different effect on growth of BC cell lines (34), to determine the net effects of 96 h-SPS–derived biomolecules on tumor growth, we also compared the tumor size in RPMI treated animals with normal saline-treated group. The size of the tumors in RPMI-treated animals was also greater than normal saline group although statistically non-significant. However, these tumors neither have any cavity nor creamy fluids as observed in 96 h-SPS solution–treated tumors, but instead, they were completely rigid on palpation (data not shown). There was no discernible change in the weights of the rats in the three groups (**Figure 4B**).

#### 3.2.2 96 h-SPS has no cytotoxic effect on animals

To check the 96 h-SPS solution toxicity on the rat, the blood index was measured in animals and no significant difference was observed (**Table 1**, *P >* 0.05). Animal organs such as liver, spleen, kidney, and lung were removed after tumor subtraction to measure their weight; however, there was no significant difference in organ weight between the three groups (**Supplementary Material Figure S5**).

**Table 1:** The blood index of cancerous rats treated with RPMI, 96 h-SPS, and normal saline.

Groups Parameter	Normal range	RPMI	96 h-SPS	Normal saline	*P*-value*
Complete blood count					
RBC (*10^6^/µl)	5.95 - 7.81	7.71 ± 0.48	7.53 ± 0.19	7.51 ± 0.3	0.58
WBC (*10^3^/µl)	1.70 - 12.15	12.89 ± 4.31	9.29 ± 1.55	7.21 ± 3.22	0.26
**MCV (fl)**	39.20 - 60.75	54.3 ± 2	53.8 ± 0.9	54 ± 1.7	0.69
**MCH pg**	20.40 - 38.15	18 ± 0.61	18 ± 0.3	18.4 ± 0.55	0.37
**MCHC (g/dl)**	35.50 - 38.20	33.1 ± 0.43	33.7 ± 0.24	13.7 ± 0.34	0.02
PLT (*10^3^/µl)	516 ± 239.84	810 ± 90.9	850.6 ± 94.2	681.5 ± 114.1	0.27
**HCT (%)**	33.60 - 44.15	41 ± 1.8	40 ± 0.72	41 ± 2	0.44
**HGB (g/dl)**	12.10 - 16.75	13.8 ± 0.76	13.6 ± 0.26	13.8 ± 0.76	0.84

Data are reported on mean ± SD.

#### 3.2.3 96 h-SPS–affected tumor metabolism-related parameters

**Figure 6:**
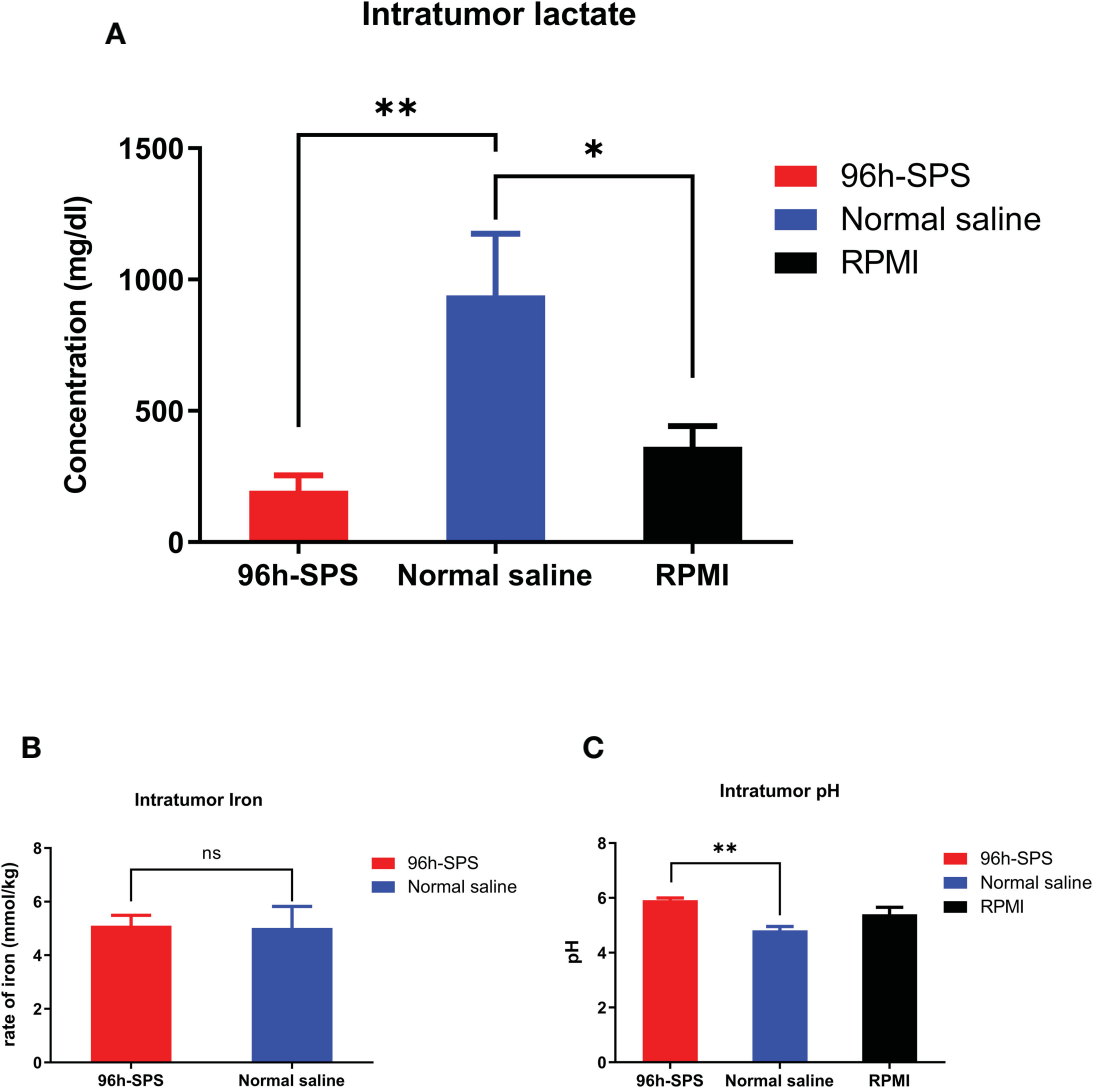
Evaluation of intra-tumor lactate, iron, and pH levels in rats tumorized with LA7. **(A)** The lactate level in different groups. The 96 h-SPS–treated cells exhibited the lowest levels of lactate **(B)** The level of intra-tumor iron in different treated groups. There was no significant difference between 96 h-SPS and normal saline in the viewpoint of iron concentration **(C)** The H^+^ ions level in all treated groups. The 96 h-SPS–treated cells exhibited the highest levels of intra-tumor pH value. Data are expressed as mean ± SD; bars indicate the SD. In each panel. ns = non-significant, *p < 0.05, and **p < 0.01.

To evaluate the effects of 96 h-SPS on LA7-induced tumor metabolism, we measured the intra-tumor lactate, iron, and pH levels in rats treated with 96 h-SPS, normal saline, and RPMI. The 96 h-SPS–treated tumors showed a significantly lower level of lactate compared with normal saline treated group (**Figure 6A**, *p<* 0.003). Although the RPMI-treated group showed an almost twofold increase in intratumor lactate compared with 96 h-SPS–treated group, but this difference was not statistically significant (*p >* 0.05). In other words, RPMI could significantly decrease the intra-tumor lactate levels compared with normal saline control group albeit with lesser strength than 96 h-SPS solution (*p =* 0.003 for 96 h-SPS *versus p =* 0.01 for RPMI alone). Reduction in H^+^ ions (decreased pH value) was significant in 96 h-SPS–treated group compared with normal saline treated group (**Figure 6C**, *p<* 0.01). This reduction was statistically not significant in the RPMI-treated group compared with normal saline control (*p >* 0.05). Intra-tumor iron level in 96 h-SPS and normal saline-treated groups was not significantly different (**Figure 6B**, *p >* 0.05).

#### 3.2.4 Downregulation of intra-tumor *Gch1*, *Spr*, *Sox2*, and *Oct4* in 96 h-SPS–treated group

**Figure 7:**
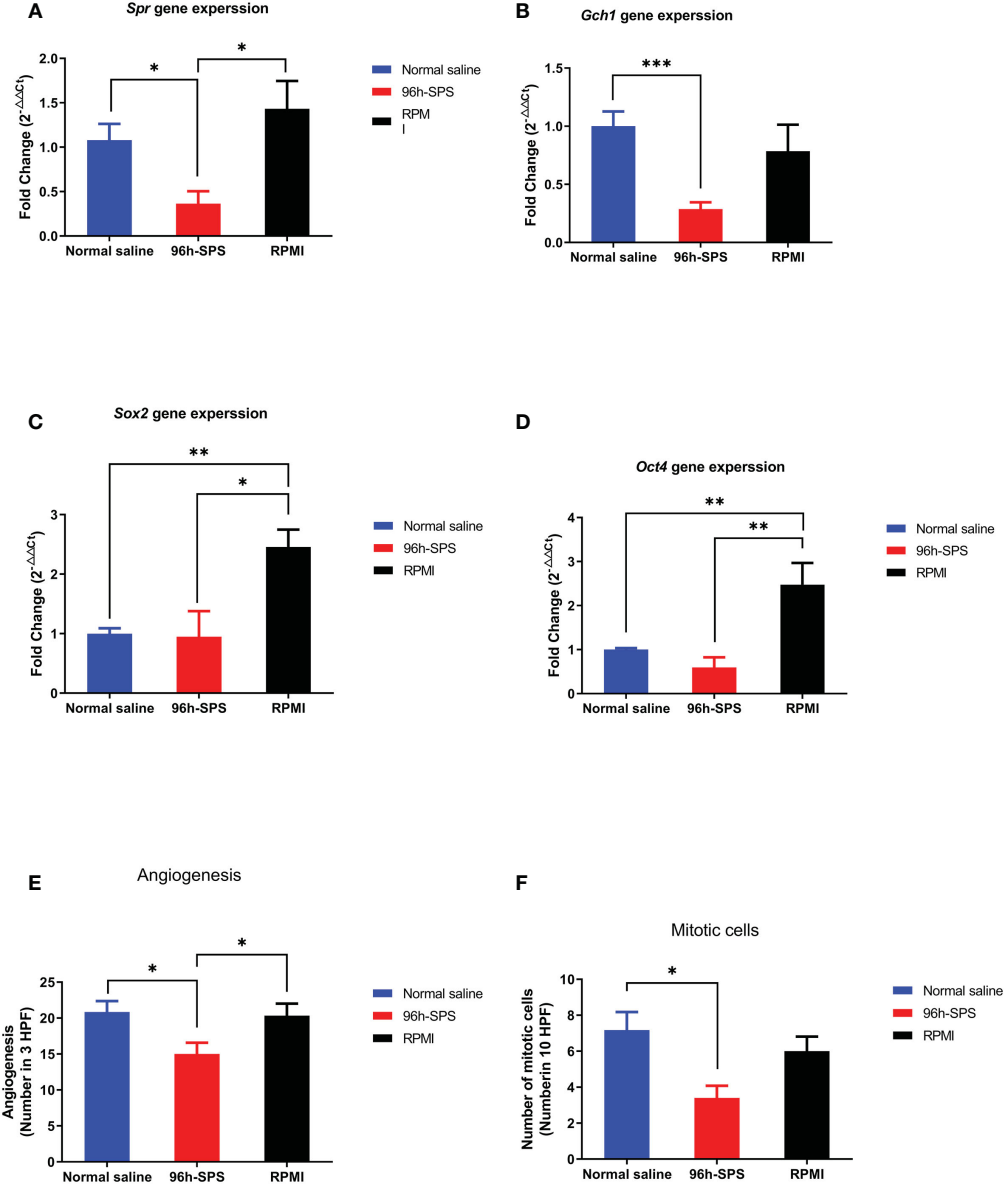
**(A–D)** Gene expression of target genes, that is, *Spr*, *Gch1*, *Oct*4, and *Sox2*. **(E, F)** Angiogenesis and mitosis in the tumors from rats exposed to 96 h-SPS, normal saline, and RPMI treatment. **(A)** *Spr* gene expression in LA7-induced tumors. In comparison with normal saline and RPMI groups, the 96 h-SPS–treated tumors showed the lowest level of *Spr* gene expression, *p<* 0.05 for both. **(B)** *Gch1* gene expression comparison between groups. 96 h-SPS but not RPMI solution was able to downregulate the *Gch1* gene expression significantly when compared with the normal saline treated tumors, *p<* 0.001 **(C)** Gene expression of *Sox2* in the tumors isolated from rats. RPMI but not 96 h-SPS solution was able to upregulate the *Sox2* expression in comparison with normal saline group, *p<* 0.05. **(D)** The expression of *Oct4* in rats treated with 96 h-SPS, normal saline, and RPMI solutions. RPMI but not 96 h-SPS solution was able to upregulate the *Oct-4* expression in comparison with normal saline group, *p<* 0.01. **(E)** The angiogenesis in three indicated groups. In comparison with two other control groups, the 96 h-SPS–treated exhibited the lowest level of angiogenesis, *p<* 0.05 for both. **(F)** The number of mitotic cells in different studied groups. In 96 h-SPS–treated tumors, but not in the RPMI group, the number of cells in the mitosis phase of the cell cycle was significantly lower than normal saline control, *p<* 0.05. Data expressed as mean ± SD, bars indicate the SD. In each panel. *p < 0.05, **p < 0.01, and ***p < 0.001.

To assess the expression of key genes involved in cell metabolism and proliferation, that is, *Spr* and *Gch1*, in LA7-induced tumors to better understand 96 h-SPS mode of action (MOA) as an anti-cancer mediator q-PCR was applied. In general, the △Ct values of the chosen four target genes varied more than the expression of *Gapdh*. The control group was used to normalize and compare the changes in the expression levels of the targets between the groups. To calculate gene expression level, 2^-△△Ct^ formula was hired for all target genes (**Figure 7**) (35). The expression level of *Spr* gene in 96 h-SPS–treated LA7-induced tumors was significantly decreased compared with RPMI or normal saline treated groups (**Figure 7A**, *p<* 0.05). The 96 h-SPS but not RPMI was able to significantly reduce the *Gch1* expression compared with the normal saline treated group (**Figure 7B**, *p<* 0.001). In addition, the expression of key genes involved in cancer differential therapy, *Sox2*, and *Oct4* was analyzed. The expression level of either *Sox2* or *Oct4* significantly increased in RPMI treated compared with 96 h-SPS or normal saline treated group (**Figures 7C, D**, *p<* 0.05).

#### 3.2.5 The histopathologic examination of LA7-induced tumors

After removing the tumor from the rat body, the middle part of all tumors was dissected with a scalpel, the pus-like liquid was drained away from 96 h-SPS–treated tumors, and the tissues were processed for light microscope histo-pathological evaluation. The angiogenesis was significantly reduced in 96 h-SPS–treated group in comparison with the normal saline and RPMI treated groups (**Figure 7E**, *p =* 0.024, and *p =* 0.037, respectively). The mitotic cell number in 96 h-SPS–treated tumors was similarly significantly lower than normal saline treated group, but not in RPMI group (**Figure 7F**, *p >* 0.05).

**Figure 8:**
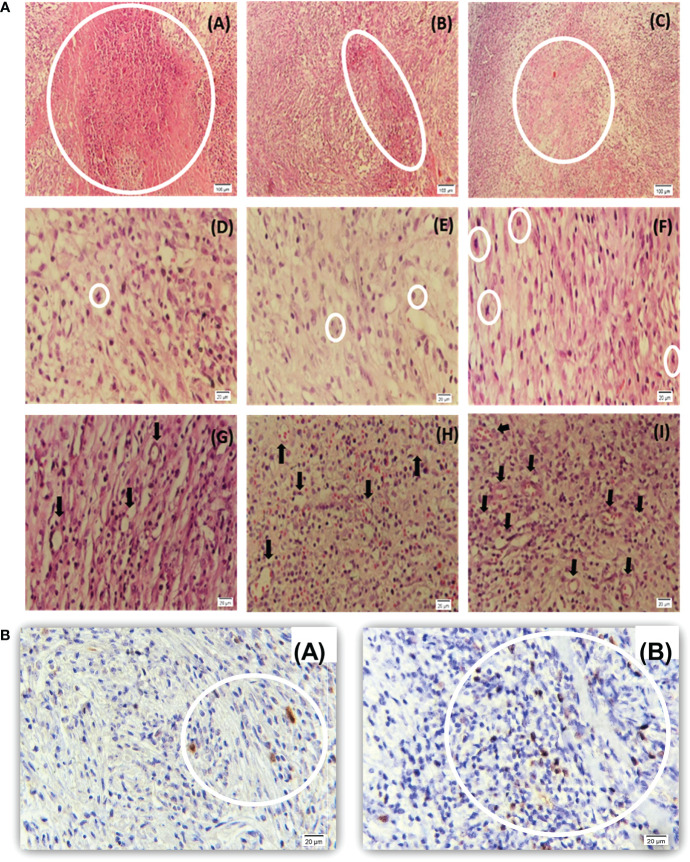
**(a)** Histopathology of LA7-induced tumors. **(A–C)** Higher necrotic area in 96 h-SPS–treated tumors **(A)** compared with the RPMI **(B)** and normal saline groups **(C)**. **(D–F)** The mitotic cell number was reduced in 96 h-SPS–treated tumors **(D)** in comparison with RPMI **(E)** and normal saline **(F)** groups. **(G–I)** Angiogenesis in three indicated groups. 96 h-SPS–treated tumors exhibited the lowest level of angiogenesis **(G)** when compared with the RPMI **(H)** or normal saline control **(I)**. *The magnification of images: **(A–C)** 10X, **(D–I)** 40X. **(b)** *Ki67* immunostained tumor–induced by LA7 cells. **(A)** Ki67-positive cells in the tumor treated with 96 h-SPS. **(B)** More Ki67-positive cells were observed in the tumor treated with normal saline in comparison with 96 h-SPS solution. *The magnification of images: **(A, B)** 40X.

Preformed H&E-stained slides inferred that the tumors treated with 96 h-SPS had extensive necrosis; nevertheless, less angiogenesis was observed compared with either RPMI or normal saline-treated groups. An increased trend of focal necrosis was observed in 96 h-SPS–treated rats in comparison with either normal saline or RPMI-treated groups (**Figures 8-[a] A–C**). **Figures 8-(a) D–F** shows a significantly lower mitotic cell number in 96 h-SPS–treated tumors compared with normal saline-treated group, whereas angiogenesis was significantly reduced in 96 h-SPS–treated rats compared with normal saline and RPMI-treated groups (**Figures 8-[a] G–I**).

#### 3.2.6 Immunohistochemistry evaluation of Ki67 in mitotic cells

To investigate mitotic cells, Ki67 antigen was assessed as a mitotic indicator in the 96 h-SPS–treated group. The lower level of Ki67 revealed that mitosis was suppressed in the 96 h-SPS–treated groups compared with normal saline-treated group (**Figures 8-[b] A, B**). Also, the histological evaluation revealed an extensive necrosis pattern and cell infiltration in the niche of the tumor (**Supplementary Material Table S2**).

#### 3.2.7 96 h-SPS solution proteins identification

To identify the protein components of the 96 h-SPS solution, we used LC-ESI-MS/MS data for database search with the software Mascot using human sequences from SwissProt database (20 259 sequences). Peptide mass tolerance was set to 50 ppm, fragment mass tolerance was set to o.6 Da, and a significance threshold *p<* 0.05 was used. Oxidation at methionine was set as variable modification for the searches. Peptides with up to one missed cleavage site were searched. An ion score cutoff of > 26 (individual ion score greater than 26 indicate identity or extensive homology) was applied for the peptide identification. Two hundred ninety-four significantly expressed proteins were identified in the solution. To compare the protein expression level in different sample groups, the RPMI medium was set as control for comparison with the 96 h-SPS group. To calculate the protein expression ratio, the mean value of the protein ratio in each group was used (Students’ *t*-test, *p<* 0.05). To gain a global insight into the 294 differentially expressed proteins, bioinformatics tools such as Gene Ontology (ClueGO V.2.5.8) and KEGG Pathway Analysis tool using Cytoscape (V 3.9.1) were applied (**Supplementary Material Figure S6**). As a result of analysis of proteins identified and a comprehensive literature review, in the 96 h-SPS solution only five additional proteins were involved in tumor biology (**Supplementary Material Table S3**).

## 4 Discussion

The most important finding of this study was that, in 96 h-SPS–treated group, the LA7 CSCs developed soft and large tumors with a large central cavity, which seemed like an abscess filled with a creamy liquid. This macroscopic manifestation was supported by our histopathologic finding, indicating an obvious necrosis lesion and higher prevalence of polymorphonuclear cells in this type of tumor. However, this finding was ambiguous with tumor development in normal saline- and RPMI-control groups, where tumors were rigid, and had smaller or almost the same size, respectively, compared with 96 h-SPS–treated tumors. This ambiguity can be clearly explained by considering our following *in vitro* and *in vivo* findings. Interestingly, the number of blood vessels was significantly reduced in 96 h-SPS–treated tumors compared with both control groups (*p<* 0.05). It is well known that angiogenesis is a critical factor for tumor progression to meet the high demand for nutrients and oxygen required for highly metabolic active tumor cells (36, 37). Indeed, hypoxia-induced necrosis is a general finding in routine pathological evaluation. This conclusion is consistent with a clinical study reported by Vasiliki Michalaki who indicated that, using anti-angiogenesis drug (sunitinib) caused an intra-abdominal tumor-like abscess formation, which mimicked disease progression, without any pathological evidence of malignancy, in a patient with renal cell carcinoma (38). Moreover, our well-controlled *in vitro* study of 96 h-SPS solution effects on LA7 viability, provided us some convincing data to conclude that, the abovementioned abscess-like tumor was not a real mass of cancerous cells, followed by LA7 CSCs proliferation, which resulted in the development of tumor with a larger size than normal saline and RPMI controls. Here, 96 h-SPS solution strongly reduced the LA7 cells viability when compared with complete cell culture medium (*p<* 0.00001) or to RPMI (*p =* 0.004). Furthermore, *in vitro* tracking of LA7 proliferation, which was performed by CFSE proliferation assay, revealed that 96 h-SPS solution–treated cells showed a slower rate of proliferation than both complete medium and RPMI controls.

In comparison with complete medium control, RPMI-treated cells exhibited a lower rate of viability (*p<* 0.00001) in MTT assay and less proliferation rate in CFSE-based proliferation analysis, however, in a lower extent compared with 96 h-SPS solution. These findings were expected as serum plays some known and unknown pivotal roles in animal cell culture including cancer cells (39). Thus, serum deprivation could induce LA7 cell death and reduce the cell viability to almost 50% in the present study. Valdis Pirsko examined the effects of three different cell culture media including RPMI-1640 on three human BC cell lines (MCF7, SkBr3, and MDA-MB-436) and reported that formulation of growth media is an important factor for culturing of these cancer cell lines (34). Since, in the body of an animal, the injected RPMI could be mixed with the interstitial fluid containing blood-derived growth substances that can be similar to substances present in cell culture serum, practically originated data from *in vitro* study by RPMI control may not be so relevant to *in vivo* data. However, as a basal medium for 96 h-SPS, it can be considered as data to predict the net effects of biomolecules produced by human immune cells during 96h of serum starvation. Accordingly, it can be said that, in our *in vitro* study, these biomolecules appeared stronger than RPMI basal medium in the context of reduction of cancer cells viability, proliferation, and migration. Furthermore, in *in vivo* study, to deduce the pure effects of such biomolecules on different indicated variables, we used normal saline group as baseline conditions for tumor growth which can be partially mimicked by complete medium in *in vitro* study for nutrient and growth factors availability view point but not for cell network complexity. Moreover, there is some evidence in the literature that convinced us to use normal saline control in this study. For instance, Valdis Pirsko group also reported that the cell culture medium with higher vitamin content improved cell yields and growth time in the abovementioned human BC cell lines (34). The association between dietary vitamin B12 and lung cancer risk was reported by Hung N Luu (40). Atoum, Manar F reported an anti-tumor activity for combination of vitamin B12 and vitamin (41). Vitamin B12 is one of the ingredients of RPMI-1640 medium, which we survey its tumor/anti-tumor effects in literature as a representative of this medium. Finally, we already reported that the DMEM-treated LA7 induced tumor in rats showed a slightly larger size compared with the normal saline group (30). Interestingly, in line with *in vitro* SFSE-based data, 96 h-SPS solution but not RPMI alone treated tumors exhibited a reduced level of mitotic cells at significant levels when compared with tumors isolated from normal saline treated tumors (*p<* 0.05). The proliferation marker, nuclear Ki 67 antigen is used as an early predictor of BC treatment efficacy and as a prognostic factor of long-term outcome for this cancer (42).Our confirmatory data obtained from Ki 67 antigen IHC staining obviously support an anti-proliferation potential for 96 h-SPS solution. Collectively, the above described data showed that 96 h-SPS solution, but not RPMI, was able to reduce the tumor angiogenesis and inhibited LA7 cell proliferation at statistically significant levels (*p<* 0.05). Certainly, further study is required to better understand the MOA of the 96 h-SPS solution in this context. However, in the present study, we found some evidence implicating that 96 h-SPS solution governs the expression of some key genes in cancer cells survival, metabolisms, proliferation, stemness, and angiogenesis. Targeting the metabolism of CSCs or pushing these cells to differentiate into mature cancer cells may therefore lead to effective treatment approaches. For instance, Xiaojing Zhan showed that the knockdown of *SPR* gene in human BC cell lines suppresses the cell’s proliferation by inducing ROS-mediated apoptosis (43). In the present study, the intra-tumor expression of *Spr* gene in 96 h-SPS–treated group was significantly lower than RPMI and normal saline groups (*p<* 0.05 for both). In comparison with the normal saline group, *Spr* gene expression was not reduced significantly in the RPMI-treated group. In the cell culture model, 96 h-SPS solution was also able to reduce *Spr* gene expression in LA7 cells much more strongly than RPMI (*p =* 0.0006) and normal saline (*p<* 0.0001). RPMI was not able to execute any significant change in *Spr* gene expression *in vitro* when compared with normal saline control. This means that the observed effect came from some unknown 96 h-SPS–specific biomolecules, which were produced during the immune cells starvation process. Moreover, GCH1 inhibition has been shown to prevent the production of BH4 and suppress tumor development *via* directly killing tumor cells and inhibiting angiogenesis (44). In addition, it improves immune response by altering proangiogenic M2 to M1 macrophages in the tumor microenvironment (44). Interestingly, the intra-tumor *Gch1* gene expression was strongly inhibited in 96 h-SPS–treated tumors, but not in RPMI group, when compared with the normal saline control group (*p<* 0.001). By taking into account that *Gch1* is a rate-limiting enzyme in BH4 production by *Spr* gene-encoded enzyme, that is, sepiapetrin reductase (45), it can be concluded that *Gch1-Spr* gene expression axis might be targeted in 96 h-SPS–treated tumor, but not in RPMI group, when the expression of *Gch1 and Spr* genes in these groups was compared with normal saline reference group. One of the main limitations of this study is that the level of BH4 was not determined in different groups neither in cell culture study nor in tumors. However, there is evidence in the literature that strongly supports the correlation between *Gch1* gene suppression and reduction of cellular BH4 levels. Jade Bailey reported that knockdown of GTPCH at the level of > 90% resulted in marked reduction in cellular BH4 level and induction of ROS generation in the mitochondria of endothelial cells (46). Besides acting as cofactors for different amino acid hydroxylase, BH4 is also a cofactor for different NOS, that is, nNOS, eNOS and iNOS, and thus promoting NO production in cells (45). NO is considered as a master regulator in cancer development and progression (47). However, in the absence or reduced levels of BH4, uncoupled NOS produces ROS instead of NO (48). ROS is an inducer of cancer cells death with different patterns including necrosis (49). *Gch1*-*Spr-*BH4 and NO/ROS imbalance axis should be examined in 96 h-SPS–treated tumors in the future, as a possible molecular mechanism involved in the induction of tumor cells death and inhibition of tumor development in the present study. On the other hand, BH4 plays an important role in iron metabolism and mitochondrial bioenergetics (50). Iron consumption and storage are increased in cancer cells, which can alter the cancer cell proliferation and migration rate, resistance to ferroptosis type of cell death, and immune escape (43).

To obtain an overall view of the intra-tumor iron levels, we compared the total iron in 96 h-SPS–treated tumor to normal saline reference group, where we saw a significant reduction in gene expression of *Gch1*-*Spr* axis. We were not able to find any significant difference in iron levels between the two groups. Moreover, we did not evaluate the intra-cellular or more especially intra-mitochondrion iron levels to provide more details. Further study at cellular and subcellular levels is needed to examine the *Gch1-Spr-BH4* axis and iron ferric/ferrous types in 96 h-SPS–treated tumors.

Surprisingly, our *in vitro* data showed that RPMI, but not 96 h-SPS, was able to enhance *Gch1* expression in LA7 cells culture; however, it lost this ability in LA7-induced tumors microenvironment whereas gained the ability to increase *Oct4* and *Sox2* stemness related gene expression. In contrast, 96 h-SPS, but not RPMI, supported the *Oct4* and *Sox2* genes expression *in vitro*, whereas these supports were lost in the tumor’s microenvironment. It is difficult to explain these controversial findings between *in vitro* and *in vivo* study by the present study; however, based on a published paper, LA7 cells as estrogen responsive CSCs can produce a solid tumor with heterogeneous population of differentiated and undifferentiated, low proliferative and high proliferative cells when injected orthotopically in rat mammary gland fat pad (51). In the present study, we also injected LA7 cells into sexually mature female rats orthotopically and the time course for tumor development was almost similar to tumor growth time course in the previously reported study, likewise, the tumors appeared around 5–6 days post-injection and reached their maximum size after 12 days. Thus, we hypothesized that the above described difference between *in vitro* and *in vivo* data may originate from some hormone such as estrogen, which was only available *in vivo*. Further study needs to establish a mechanism underpinning this difference. However, it seems logical if we explore such mechanisms in RPMI-treated LA7 cells *in vitro* and *in vivo*. This medium is formulated with different vitamins such as B12 and antioxidant substances such as reduced form of glutathione and glucose, which can play pivotal roles in cancer cell metabolism and differentiation. Lactate, which is an important substance produced by glucose metabolism, is considered as an important biomolecule in different aspects of tumor biology study. In the present study, we measured the intra-tumor levels of lactate and pH. We showed that, in association with significant levels of increased pH in 96 h-SPS–treated tumors, the lactate level was reduced significantly, but the tumors treated with RPMI could reduce the lactate production without affecting pH value significantly when compared with reference normal saline control. It means that RPMI could have some *in vivo* effects on cancer cells metabolism and growth as previously reported in *in vitro* study (34).

Finally, the difference in executing anti-cancer biological functions between 96 h-SPS and its basal medium, RPMI, was well documented in our LA7 cells migration assay. Scratch wound healing is used to predict the anti-tumor migration activity of indicated molecules (52). As **Figure 2** shows, the initial empty area created by scratch was linearly decreased in both completed medium and RPMI controls, whereas this area was linearly increased in 96 h-SPS–treated cells. This finding confirmed the result of the MTT assay that proved the LA7 cytotoxicity effect of 96 h-SPS and CFSE based data indicated the anti-cancer cell proliferation activity for this solution.

Moreover, we hypothesized that a specific type or a group of proteins in 96 h-SPS might be involved in affecting tumors. To elucidate the exact effector element of the 96 h-SPS treatment, we analyzed the 96 h-SPS protein composition, in which Hsp60, RhoGDI2, CXCL5, and SPARC were discovered.

Hsp60 was one target protein, which was found in 96 h-SPS proteins identified by proteomics analysis. Hsp60 has different roles in immune response such as secretion of cytokines from professional antigen-supplying cells and activates T cells (53). It can also stimulate dendritic cell maturation through TLR2-4 signal transduction pathways and synergize IFN-γ proinflammatory action (54). Another protein that has recently been shown to suppress cancer metastasis is Rho GDP dissociation inhibitor 2 (RhoGDI2). The loss of RhoGDI2 expression in patients is closely correlated with cancer pathology and progression of metastasis (55). It has been observed that the NT-domain of SPARC and its 51-aa peptide are very effective in modulating and increasing apoptosis, thus increasing the sensitivity to chemotherapy drugs in resistant tumors, which provides more insight into the mechanisms of resistance to chemotherapy (56). CXCL5 is a chemokine that stimulates the chemotaxis of neutrophils and plays an active role in recruiting different immune cells into inflammatory sites (57). Furthermore, we discovered that PMNCs were the most common migratory cells in 96 h-SPS–treated tumors with a mixed pattern of inflammatory cell migration, such as mast cells.

It would be beneficial to study the behavior of immune cells in the microenvironment of solid tumors under favorable conditions, such as under starvation stress, which they grow under growth factor deprivation, to gain a better understanding of how tumors are treated. Additionally, cancer and immune cells are exposed to nutrient challenges in the tumor microenvironment; a model such as serum starvation conditions can be a suitable *in vitro* model to research tumor metabolism biology.

## 5 Conclusions

Taken together, the data generated in this study indicate that 96 h-SPS solution–derived biomolecules may help to prevent BC stem cell–mediated tumor development and progress. This effect could be exerted through different mechanisms such as direct cytotoxicity, inhibition of cell proliferation and migration, in association with reduction in *Gch1* and *Spr* genes expression, mitigation of angiogenesis and mitosis, and increasing of necrosis. The preliminary data obtained from the present study need to be investigated on a larger scale and can be used as a pilot for further studies on the biology of cancer development.

## Data availability statement

The mass spectrometry proteomics data have been deposited to the ProteomeXchange Consortium via the PRIDE (58) partner repository with the dataset identifier PXD039472.

## Ethics statement

The animal study was reviewed and approved by the local ethical committee under reference number IR.MUBABOL.HRI.REC.1398.221 at Babol University of Medical Sciences.

## Author contributions

Conceptualization: AM, BK, RG; Methodology: MM, MR, HN, HA-N, SF; Investigation: MM, MR, HK, SF, KN, MR, HHN; Formal analysis: DS, RG, AM; Resources: BK, AM; Data curation: BK, RG, AM; Writing-original draft preparation: MM, RG; Writing-review and editing: RG, AM, BK; Visualization: RG; Supervision and project administration: BK, AM. All authors contributed to the article and approved the submitted version.
