# Supplementary material for: Ninety-six–hour starved peripheral blood mononuclear cell supernatant inhibited LA7 breast cancer stem cells induced tumor via reduction in angiogenesis and alternations in Gch1 and Spr expressions
Source: Front Immunol. 2023 Feb 23;13:1025933. doi: 10.3389/fimmu.2022.1025933 (PMC9996193; doi:10.3389/fimmu.2022.1025933)
Supplement: Supplementary file 1 [file DataSheet_1.pdf]

Table S1: Types and Characteristics of primers used in this study

Gene	Sequence 5'→3'
**GCH1**	**Specific forward primer:** GCCGCTTACTCGTCCATCCTG
	**RT-PCR primer:** GTCGTATCCAGTGCTGCGACCGTATGGATGTGTCTGCGGC GTTTTATCATGCACTGGATACGACTCACCATCTCGTC
**SPR**	**Specific forward primer**: GCTGTTGAGGAACCCAGTGTGAG
	**RT-PCR primer**:GTCGTATCCAGTGCTGCGACCGTATGGATGTGTCTGCGGCGTT TTATCATGCACTGGATACGACCACAGTCCACCAG
**GAPDH**	**Specific forward primer**: CAGTGCCAGCCTCGTCTCATAG
	**RT-PCR primer:** GTCGTATCCAGTGCTGCGACCGTATGGATGTGTCTGCGGCGTTTTATCA TGCACTGGATACGACCGTTCACACCGA
**Sox2**	**Specific forward primer**: CCACCAATCCCATCCAAATTAACG
	**RT-PCR primer**: GTCGTATCCAGTGCTGCGACCGTATGGATGTGTCTGCGGCGTTTTATCA TGCACTGGATACGACTCCCTGCGAAG
**Oct4**	**Specific forward primer**: TCCCGAGGAGTCCCAGGATATG
	**RT-PCR primer** GTCGTATCCAGTGCTGCGACCGTATGGATGTGTCTGCGGCGTTTTATCA TGCACTGGATACGACGGCAGATGGTTG

Table S2: histological variables and infiltrated cells in LA7 induced tumors.

**Groups**	**Rat number**	**Inflammatory cell infiltration**	***Necrosis**	**Mast cell**
**RPMI**	1	Lymphocyte	-	+
	2	Lymphocyte	+	+
	3	Lymphocyte	+	+
	4	Lymphocyte	-	+
	5	Lymphocyte	-	-
	6	Lymphocyte	-	-
**96h-SPS**	1	PMNs	+	+
	2	PMNs	+	+
	3	PMNs	+	-
	4	Lymphocyte	+	-
	5	Lymphocyte	+	-
**Normal saline**	1	Lymphocyte	-	+
	2	Lymphocyte	+	+
	3	Lymphocyte	-	+
	4	Lymphocyte	+	+
	5	Lymphocyte	+	-
	6	Lymphocyte	-	+

* (+) and (-) symbols indicate, “above 50% necrosis” and “below 50% necrosis” respectively. PMNs: Polymorphonuclear cells.

Table S3: Data related to five proteins expected to be involved in the anticancer effect and necrosis induction, derived from an analysis of 96 h-SPS solution by Mass spectrometry.

**Number**	**Accession**	**Protein name**	**Protein score**	**Protein mass**	**Protein Coverage**
**1**	A1AT_HUMAN	Alpha-1-antitrypsin	62	46878	4.5
**2**	EMIL1_HUMAN	EMILIN-1	34	107941	0.8
**3**	GDIR2_HUMAN	Rho GDP-dissociation inhibitor 2	235	23031	18.9
**4**	SPRC_HUMAN	SPARC	160	35465	15.8
**5**	CH60_HUMAN	60kDa heat shock protein, mitochondrial	67	61187	2.1

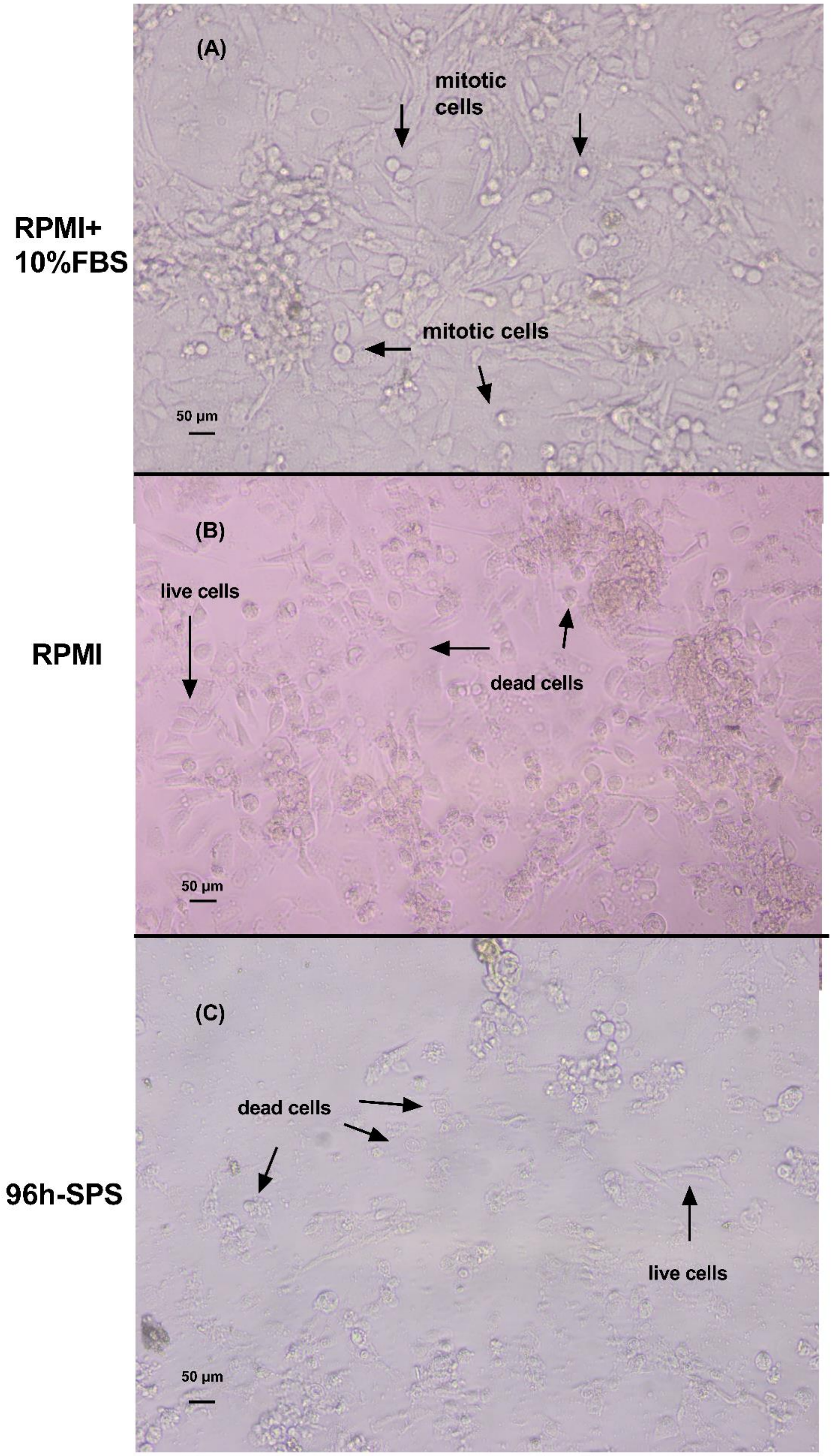
RPMI+
10%FBS
(A)
mitotic
cells
mitotic cells
50 µm
RPMI
(B)
live cells
dead cells
50 µm
96h-SPS
(C)
dead cells
live cells
50 µm

Figure S1: Morphology of LA7 cells after 24 h incubation with complete medium, RPMI, and 96 h-SPS. **A)** Complete medium with shine cells and spindle-shaped cells, **B)** RPMI containing round nucleus and dying cells, **C)** 96 h-SPS with high prevalence of dying cells.

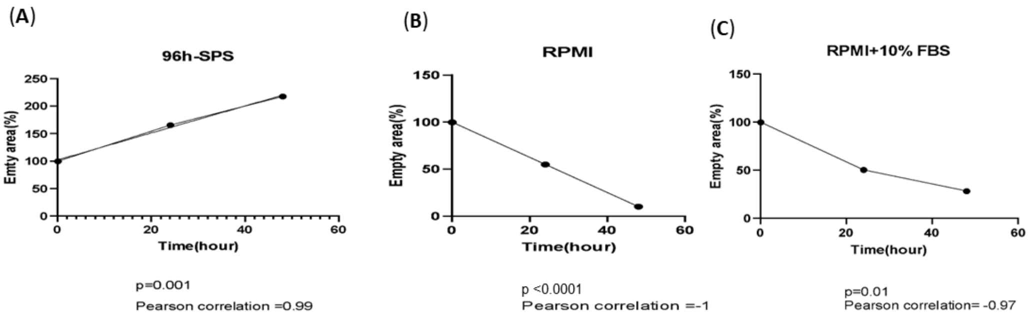

Figure S2: Correlation between time and empty area (%) in the scratch test. **A)** In 96 h-SPS group r- value was +0.99, p<0.001. **B)** In RPMI there was a maximum level of inverse correlation between time and empty area, r=-1, p<0.0001. **C)** In complete medium control groups similar to RPMI group correlation between time and the empty area was strongly negative r=-0.97, p=0.01.

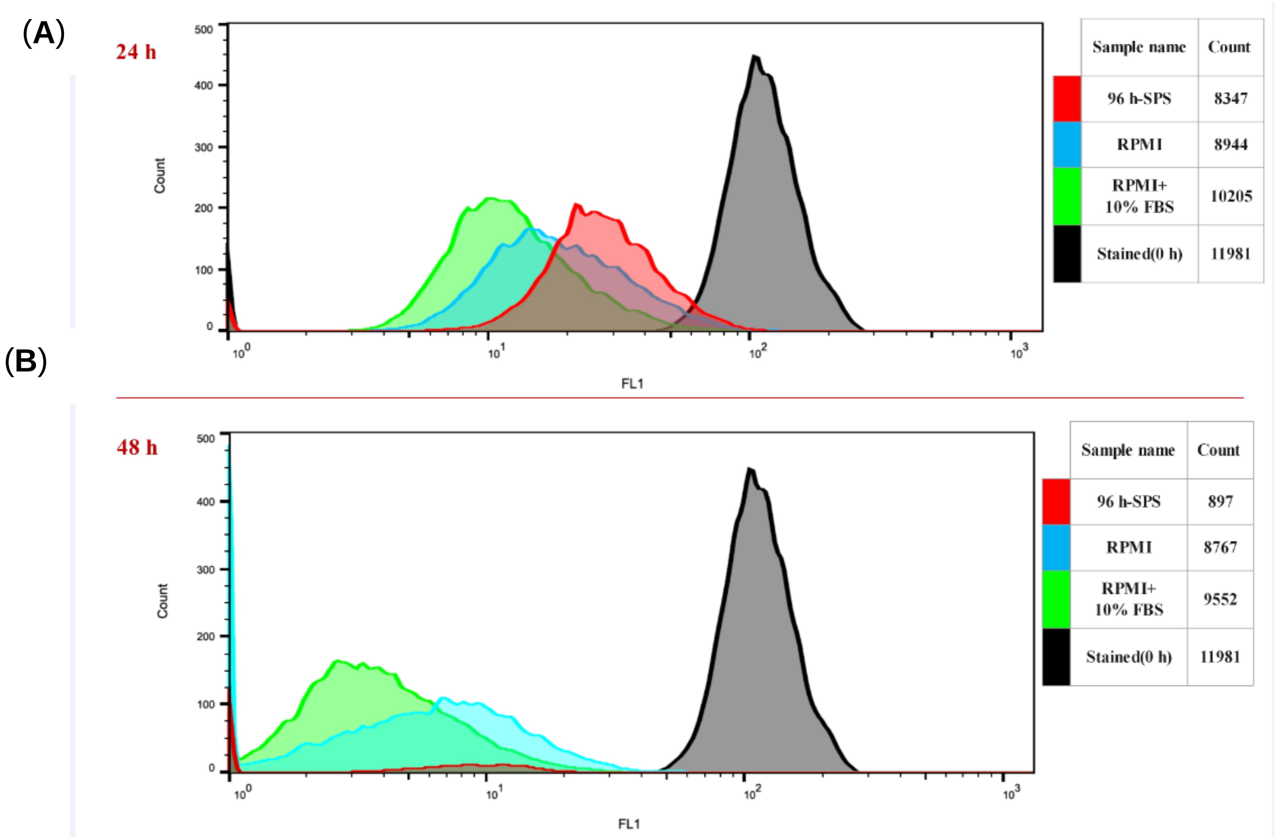

Figure S3: Proliferation test with CSFE of LA7 cells exposed to complete medium, RPMI and 96 h-SPS solutions. **A)** LA7 cells were stained with CFSE after 24 h incubation, the cells were treated with different solutions and were analyzed with flow cytometry for 24 h and 48 h later. CFSE fluorescence histogram shows cells that were exposed to 96 h-SPS after 24 h, postponed the cell proliferation in comparison to complete medium or RPMI treated cells. **B)** After 48 h, the number of 96 h-SPS treated SFSE positive cells in comparison with other groups sharply decreased.

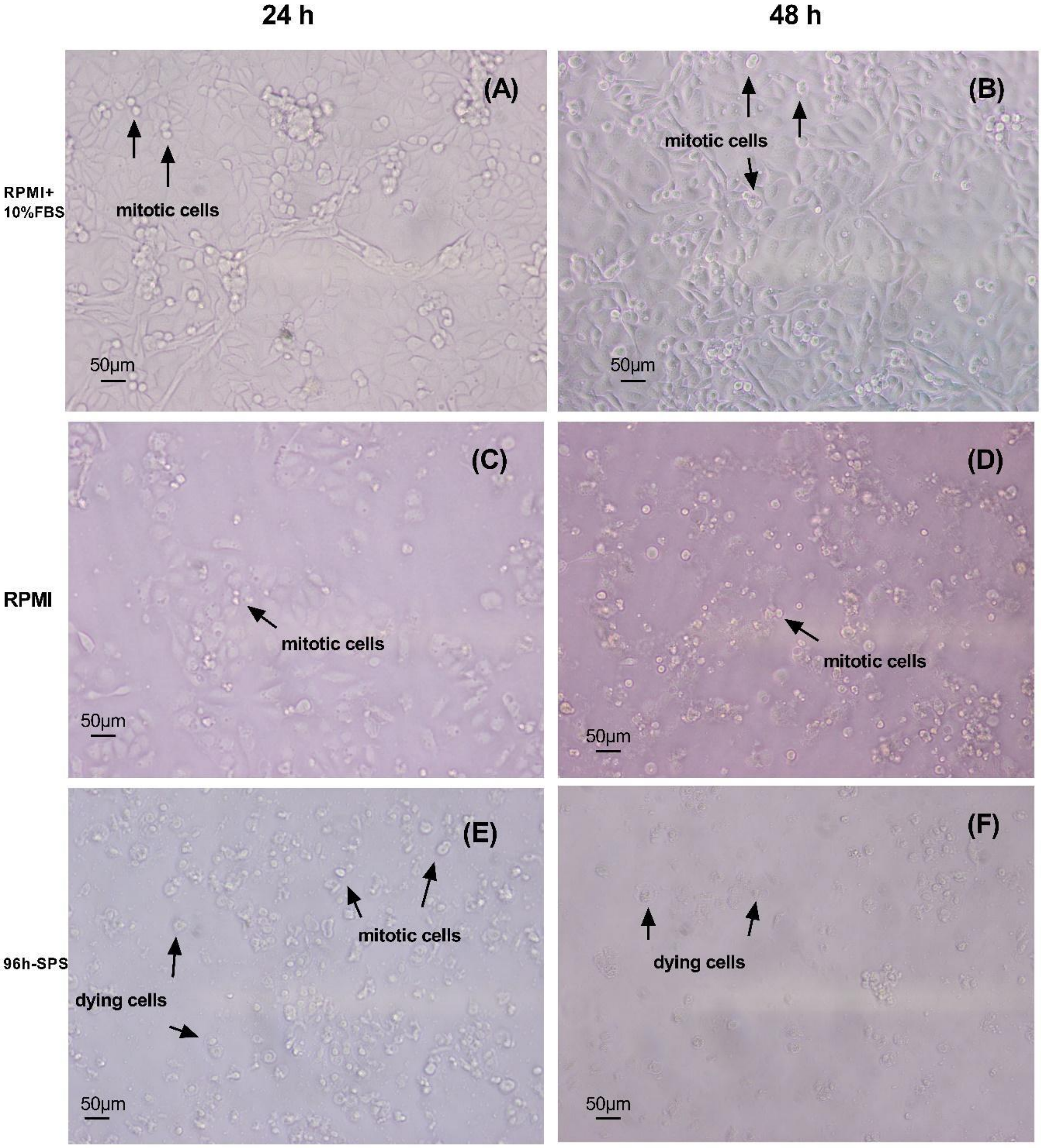
24 h
48 h
RPMI+
10%FBS
(A)
mitotic cells
50µm
(B)
mitotic cells
50µm
RPMI
(C)
mitotic cells
50µm
(D)
mitotic cells
50µm
96h-SPS
(E)
mitotic cells
dying cells
50µm
(F)
dying cells
50µm

Figure S4: Morphology of CFSE stained LA7 cells treated with indicated solutions. After 24 h of cell incubation, the dying cells appeared in 96 h-SPS treated cell populations (arrows in panel **E**) and the cell density was obviously decreased when compared to the complete medium and RPMI control cells (panels **A** and **C** respectively). However, some mitotic cells were also observed in this group (arrows in panel **E**). After 48 h, the empty area in 96 h-SPS treated cells was increased (panel **F**) while the density of the cells increased in the complete medium-treated group which was accompanied with mitotic cells (arrows in panel **B**). After 48 h of cell incubation with RPMI alone, some cells were at the mitosis phase of the cell cycle (panel **D**). ***The magnification of images: 40X.

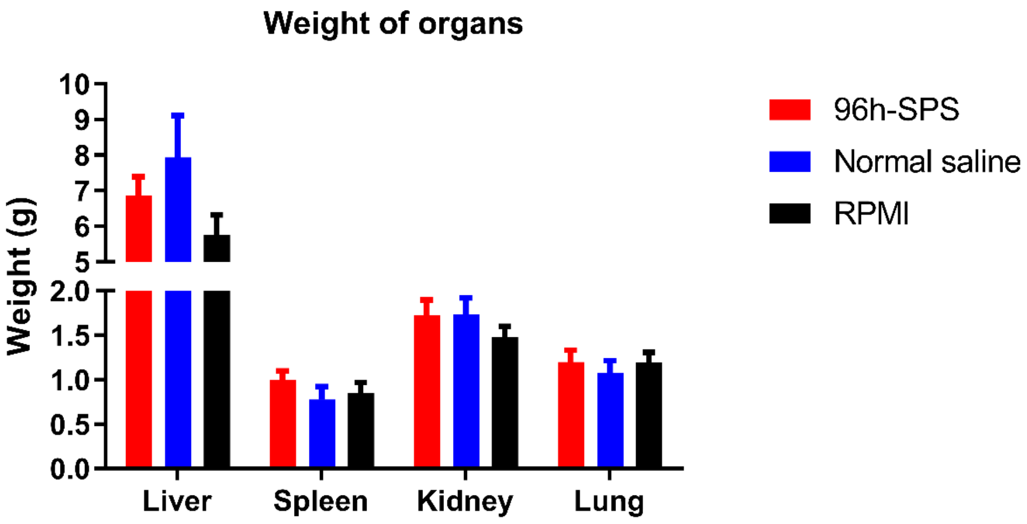

Figure S5: The organ weight in the cancerous rats treated with 96h-SPS, RPMI, and Normal saline. There was not any significant difference between 96 h-SPS treated animals and two control groups in weight of indicated organs.

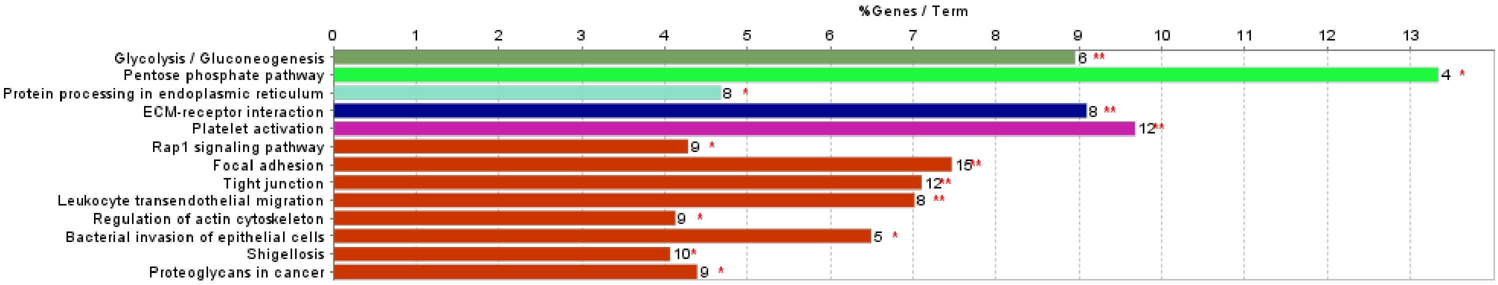

Figure S6: KEEG Pathway Analysis of 96-h starved PBMCs supernatant.
